# Perinatal Asphyxia: A Review from a Metabolomics Perspective

**DOI:** 10.3390/molecules20047000

**Published:** 2015-04-17

**Authors:** Claudia Fattuoni, Francesco Palmas, Antonio Noto, Vassilios Fanos, Luigi Barberini

**Affiliations:** 1 Department of Chemical and Geological Sciences, University of Cagliari, Cagliari I-09042, Italy; E-Mail: francesco.palmas@unica.it; 2 Department of Surgical Sciences, University of Cagliari and Neonatal Intensive Care Unit, Puericulture Institute and Neonatal Section, Azienda Ospedaliera Universitaria, Cagliari I-09042, Italy; E-Mails: antonotocagliari@gmail.com (A.N.); vafanos@tiscali.it (V.F.); 3 Department of Public Health Clinical and Molecular Medicine, University of Cagliari, Cagliari I-09042, Italy; E-Mail: barberini@unica.it

**Keywords:** metabolomics, asphyxia, perinatal, brain, blood, urine, early diagnosis, biomarkers, NMR, mass spectrometry

## Abstract

Perinatal asphyxia is defined as an oxygen deprivation that occurs around the time of birth, and may be caused by several perinatal events. This medical condition affects some four million neonates worldwide per year, causing the death of one million subjects. In most cases, infants successfully recover from hypoxia episodes; however, some patients may develop HIE, leading to permanent neurological conditions or impairment of different organs and systems. Given its multifactor dependency, the timing, severity and outcome of this disease, mainly assessed through Sarnat staging, are of difficult evaluation. Moreover, although the latest newborn resuscitation guideline suggests the use of a 21% oxygen concentration or room air, such an approach is still under debate. Therefore, the pathological mechanism is still not clear and a golden standard treatment has yet to be defined. In this context, metabolomics, a new discipline that has described important perinatal issues over the last years, proved to be a useful tool for the monitoring, the assessment, and the identification of potential biomarkers associated with asphyxia events. This review covers metabolomics research on perinatal asphyxia condition, examining in detail the studies reported both on animal and human models.

## 1. Brief Introduction to Perinatal Asphyxia

Perinatal asphyxia is defined as an oxygen deprivation that occurs around the time of birth, and may be caused by perinatal events such as maternal or foetal haemorrhage, intermittent or acute umbilical cord compression, uterine rupture or shoulder dystocia, influencing the supply of oxygenated blood to the foetus. This medical condition affects some four million neonates worldwide per year, causing the death of one million subjects [1]. In most cases, infants successfully recover from hypoxia episodes, however, some patients may develop ischemic encephalopathy (HIE), leading to permanent neurological conditions such as seizure disorders, cerebral palsy, cognitive delays, and motor disabilities. Furthermore, asphyxia and ischemia are responsible for the impairment of different organs and systems (central nervous system 28%, cardiovascular system 25%, kidneys 50%, and lungs 23%) [2]. Since the availability of molecular oxygen is compromised, HIE seems to involve the terminal electron acceptor in oxidative phosphorylation (OXPHOS) and the reduction of ATP and reactive oxygen species (ROS). These variations lead to depolarization of the mitochondrial membrane and increment of intracellular Ca^2+^, hence inducing apoptosis. Moreover, neurological injuries may derive from glutamate-mediated excytotoxicity and failure of the ATP-dependent ion pumps. Therefore, given its multifactor dependency, the severity and outcome of this disease are of difficult evaluation. Indeed, the clinical diagnosis for asphyxia considers several criteria with the two most important being the occurrence of cardiorespiratory or neurological depression, and acute hypoxia in the presence of acidemia [3]. Moreover, the oxygen concentration for neonatal resuscitation is still under debate [4]. To our knowledge, the latest guideline concerning newborn resuscitation was published in 2010 and it suggests the use of a 21% oxygen concentration or room air for the initial resuscitation in neonates > 32 weeks gestation, and oxygen-enriched air in smaller ones, ideally guided by pulse oximetry [5,6,7]. However, the pathological mechanism is still not clear, and a golden standard treatment to reduce the potential impairments has yet to be defined. Currently, the timing and severity of asphyxia are mainly assessed through Sarnat staging, which relies on clinical parameters to classify infant encephalopathy as mild, moderate or severe [8]. Since more accurate biomarkers are necessary for early therapeutic interventions, several studies have applied magnetic resonance (NMR) spectroscopy and imaging, electroencephalography [9], and target biofluid (blood, CSF) analysis focused on certain molecules to investigate the possibility of a quantitative approach. Unfortunately, no compound or combination of compounds has been validated yet, requiring further research on this topic. A recent strategy that may be employed for both the evaluation of resuscitation methods, and the discovery of novel biomarkers for asphyxia and HIE, leading to significant changes in clinical practice, is metabolomics.

## 2. Metabolomics

Metabolomics is the youngest “omics” discipline, and it applies a holistic approach, rather than a reductionist one, to the components and molecules such as genes, transcripts, proteins, and metabolites that constitute cells, tissues, or organisms [10]. Indeed, it aims to integrate different levels of information for a global understanding of biological systems. Metabolomic analysis of bio-fluids or tissues has already found applications in physiology, functional genomics, pharmacology, toxicology, and nutrition, highlighting the capacity of such method to recognize different groups, due to their peculiar metabolic profile [11,12,13]. In fact, the presence of endogenous metabolites in bio-fluids may describe the cellular phenotype. Moreover, through the rapid characterization of small molecules (metabolites), this new approach has the potential to explore genotype-phenotype and genotype-environment interactions, delivering an accurate snapshot of the subject’s metabolic status. In this context, the aim of metabolomics is to improve early diagnosis, classification, and prediction over the development of a pathological condition. Currently, metabolomics applications and techniques are in a phase of exponential growth, and it is already clear that this strategy will play a significant role in the discovery of clinical and pharmacological biomarkers [14]. Interestingly, over the last years important studies have been reported on perinatal diseases. For instance, metabolomics proved to be a useful tool for the monitoring of pharmacological treatments for Patent Ductus Arteriosus (PDA), for the recognition of infants born with asymptomatic cytomegalovirus infection, and for the identification of potential biomarkers associated with intrauterine growth restriction [15,16,17]. Given the versatility and importance of such discipline, this review covers metabolomics research on perinatal asphyxia condition, examining in detail the studies reported both on animal and human models.

## 3. Animal Models

Animal models reported in literature from 2010 to 2014 consist of rodent, monkey, and piglet studies. Experimental design and results are described below, while the main findings are summarised in Table 1.

### 3.1. Rat and Mouse Model

In order to investigate the response of hypothermia treatment, and to characterize helpful and harmful intracellular events on eventual brain damages, Liu and co-workers [18,19,20,21] dedicated four papers on the NMR analysis of rats and mice brain tissue. In fact, having a deeper knowledge on such mechanisms, it is thought to further validate such practice and to tailor a more efficient treatment, leading to better outcomes for patients.

**Table 1:** Metabolomic analysis on animal models.

Species and Population	Method	Biofluid/Tissue	Aim	Discriminant Metabolites/Indicators	Reference
Sprague-Dawley rat 10	^1^H-NMR ^31^P-NMR	brain slice extract	Hypothermic therapy validation	**Increase in hypothermia**: ATP, ADP, PCr, *N*-acetylaspartate, glutamate, taurine **Decrease in hypothermia**: acetate, adenine, alanine, choline, isoleucine, lactate, leucine, tyrosine, valine, inosine, arginine	Liu *et al*., 2011 [18]
Sprague-Dawley rat 10	^1^H-NMR ^31^P-NMR ^13^C-NMR	brain slice extract	Hypothermic therapy validation	**Significant for clustering**: pyruvate carboxylase metabolites/pyruvate dehydrogenase metabolites ratio, acetate/glucose ratio **Increase in hypothermia**: taurine, PCr, glutamine **Decrease in normothermia**: glutamine	Liu *et al*., 2012 [19] Liu *et al*., 2013 [20]
Mice 6 hypothermia, no reoxygenation 6 controls 6 hypothermia 6 normothermia 6 hypothermia, rewarming 6 long normothermia	^1^H-NMR	whole brain extract	Hypothermic therapy validation	**Increase in hypoxia**: alanine, ADP, choline, lactate, succinate, valine, γ-aminobutyric acid, isoleucine **Decrease in hypoxia**: ATP, PCr, phosphocholine (PCho), malate, aspartate, taurine, *N*-acetylaspartate **Increase in normothermia**: fumarate, succinate, isoleucine, *N*-acetylaspartylglutamate, acetate, formate **Decrease in normotermia**: taurine, histidine, malate, ascorbate **Significant for clustering**: taurine, acetate, formate, fumarate, glutamine, *N*‑acetylaspartylglutamate, succinate.	Liu *et al*., 2013 [21]
Macaca nemestrina20 umbilical cord clamping *vs.* controls	GC × GC-TOFMS	umbilical cord blood	Differences between pre- and post-asphyxia	**Increase**: succinic acid, lactate, glutamate, 9H-purine, malate, glycerol, glucose, arachidonic acid, leucine, creatinine, fructose, myo-inositol, butanoic acid, pantothenic acid	Beckstrom *et al*., 2011 [22]
Macaca nemestrina 6	GC × GC-TOFMS	blood	Metabolites change from foetus to neonate	**Increase**: glutamic acid, myo-inositol, α-ketoglutaric acid, fumaric acid, malate, succinyl CoA, propanoic acid, d-ribohexitol, erythro-pentonic acid, gluconic acid lacton, glucuronic acid, glucose-1-phosphate, maltose, isovaleric acid, tryptophan, butanoic acid, creatinine, threonic acid, xylose **Decrease**: lactate, proline, hippuric acid, benzoic acid	Beckstrom *et al*., 2012 [23]
Piglets 27	(FIA)-MS/MS LC-MS/MS	urine	Resuscitation response to different oxygen concentrations	**Increase in hypoxemia**: lactate, α-ketoglutarate, succinate, fumarate **Increase with 100% oxygen resuscitation**: lanosterol, 24-*S*-hydroxycholesterol, 25-hydroxycholesterol **Increase in severe hypoxemia**: long chain acyl carnitines **Decrease in severe hypoxemia** carnitine, decadienyl-l-carnitine	Solberg *et al*., 2010 [24]
Piglets 38	NMR	urine	Resuscitation response to different oxygen concentrations	**Increase in 18% oxygen**: carbohydrate metabolism **Increase in 21% oxygen**: cellular function maintenance. glucose, lactate, alanine, glyceric acid, pyruvic acid, malonic acid, glycine, succinate, 3-methyladenine, acetylglycine, glutaconic acid, 4-hydroxyphenylpyruvic acid, 3-hydroxymethylglutarate **Increase in 40%–100% oxygen**: free radical processes.creatinine, urea, citric acid, tartaric acid, ethanol, glucose, indoxyl sulphate	Fanos *et al*., 2014 [25]
Yorkshire-Landrace piglets 17 hypoxia *vs.* 15 controls	NMR	urine	Recognition hypoxia *vs.* control	**Increase**: 1-methylnicotinamide, 2-oxoglutarate, asparagine, betaine, citrate, creatine, fumarate, lactate, *N*-acetylglycine, *N*-carbamoyl-β-alanine, valine **Decrease**: alanine, hippurate	Skappak *et al*., 2013 [9]

In 2011 [18], an *ex vivo* study was conducted on 20 cerebrocortical slices from 10 anesthetized 7-day-old Sprague-Dawley rat littermates of either sex. During the experiment, neonatal rat brain slices, placed in a superfusion chamber containing fresh, oxygenated artificial cerebrospinal fluid (oxy-ACSF), underwent 45 min of oxygen-glucose deprivation (OGD) through replacement of the oxy-ACSF with deoxygenated and glucose-free ACSF. Successively, the excised tissues were collected at different time points and divided into three different groups. One of the groups was subjected to mild hypothermia conditions (32 °C) for 3 h, while another group was treated with the same conditions after a delay of 15 min. In both cases, hypothermia was followed by 3 h of normothermic (37 °C) recovery. The remaining slices served as normothermic control group (6 h). Subsequently, tissues were frozen, grounded, extracted and resuspended in D_2_O.

Samples were then analyzed through ^1^H- and ^31^P-NMR. Although the NMR platform was able to identify 49 metabolites in total, the discriminant molecules, after Principal Component Analysis (PCA) and Partial Least Square Discriminant Analysis) PLS-DA multivariate analysis, were found to be six. In particular, when compared to the normothermia group, higher concentrations of ATP, ADP, phosphocreatine (PCr), *N*-acetylaspartate, glutamate, and taurine were observed in the hypothermia and delayed hypothermia, while acetate, adenine, alanine, choline, isoleucine, lactate, leucine, tyrosine, and valine levels decreased within the same samples (inosine and arginine decreased only in the hypothermia group). Moreover, enzyme-linked immunosorbent assay (ELISA) cell-death quantification, and superoxide production quantification were performed on the samples to assess cell death rate. Such techniques showed a decrease in the death rate for the treatment with immediate hypothermia compared with normothermia and delayed hypothermia cases. Therefore, metabolomics NMR approach allowed for the distinction between normo- and delayed hypothermia where traditional essays could not deliver such a fine data. In 2012 and 2013, Liu’s group reported two similar studies on rat brain slices subjected to a 4 °C difference hypothermia [19,20]. Following [1-^13^C] glucose and [1,2-^13^C] acetate administration, tissue extracts were analysed through ^1^H-, ^31^P- and ^13^C-NMR techniques, adding information regarding Krebs cycle changes. Further statistical analysis led to the identification of (pyruvate carboxylase)/(pyruvate dehydrogenase) activity, and acetate/glucose ratios as important biomarkers for asphyxia conditions. In fact, comparing the three experimental groups, pyruvate carboxylase/pyruvate dehydrogenase ratio resulted higher in hypothermic samples, followed by delayed hypothermic and normothermic cases. This occurrence, which is useful for ATP preservation, may be explained due to the high levels of glutamine and glutamate, key metabolites affected by pyruvate carboxylase activity, and their Krebs cycle precursors [4-^13^C] α-ketoglutaric acid and oxaloacetate (either 2- or 3-^13^C). Decreased glutamine concentrations were observed in normothermia group. Furthermore, other significant metabolites were [2-^13^C] taurine, and PCr. Regarding the variation of the acetate/glucose ratios, it may suggest modification in metabolite transportation (exchange between neuron-produced glutamate to astrocytes, and obtained glutamine to neurons). Therefore, the only significantly potential biomarker suggested by this study is glutamine, which increased in all hypothermia treated subjects.

The same group later designed a different experiment [21]. Indeed, after ligation of the right common carotid artery, hypoxia was induced to postnatal day 7 littermate mice of either sex through 30 min of 8% oxygen-92% nitrogen mixture flow. Following 3.5 h of either hypothermia (31 °C) or normothermia (37 °C) and eventual rewarming, mice were decapitated to obtain the whole brains. These tissues were then treated for extraction and analyzed by ^1^H-NMR. The 36 mice used for this experiment were divided into six groups: control (no hypoxia, no hypothermia); hypoxia with no reoxygenation; hypoxia and hypothermia with no rewarming; hypoxia and normothermia; hypoxia followed by hypothermia and slow rewarming (24 h after hypoxia); hypoxia and normothermia with brain collection after 24 h. For NMR analysis, two whole brains from sibling mice of the same group were processed together. According to the chemometric analysis, several considerations were possible. A clear separation for the hypoxia group from all end of recovery groups, which overlap among themselves, was allowed due to higher concentration of alanine, ADP, choline, lactate, succinic acid, valine, γ-aminobutyric acid, and isoleucine, and lower values of ATP, PCr, PCho, malate, aspartate, taurine, and *N*-acetylaspartate. Similarly, the normothermia group was characterized by an increase in glutamine, fumarate, succinic acid, isoleucine, *N*-acetylaspartylglutamate, acetate, and formate levels, while taurine, histidine, malate, and ascorbate decreased. Moreover, although no separation criteria was identified for condition immediately after mild hypothermia treatment, this study was able to distinguish normothermia from hypothermia and control groups at 24 h after hypoxia-ischemia occurrence. This identification was possible because of the higher levels of taurine (hypothermia *vs*. normothermia), and the decrease of acetate (hypothermia *vs.* normothermia, control *vs*. normothermia), formate (hypothermia *vs*. normothermia, control *vs.* normothermia), fumarate (control *vs*. normothermia), glutamine (control *vs*. normothermia), *N*-acertylaspartylglutamate (hypothermia *vs*. normothermia), and succinic acid (hypothermia *vs.* normothermia).

The advantages of these experimental designs are the accuracy of the *ex vivo* neuron/glial architecture, which is close to the *in vivo* one. Concerning the first three strategies, OGD process can be precisely controlled, but insult recovery is faster in these cases and it may depend on multiple inhibited mechanisms. Furthermore, the metabolites diffusion may be modified compared to *in vivo* environment. Conversely, the induction of hypoxia under *in vivo* conditions, may deliver a more accurate description of the pathology, involving normal metabolic pathways and diffusion systems. Nevertheless, in both designs NMR techniques cannot distinguish between a locally restricted or overall metabolite pauperization and larger data set is required for data meaning. Far from achieving a clinical application, metabolomic analysis showed its pivotal role for the distinction of different treatments and outcomes groups.

### 3.2. Macaca Nemestrina Model

Regarding non-human primate models, the species of choice was the pigtail macaque (*Macaca nemestrina*). Indeed, Beckstrom and co-workers [22] analysed umbilical blood samples while conducting two different studies. In the first work in 2011, the primates under study were subjected to hysterotomy at 5 ± 2 days prior to term, pre-birth blood samples were collected by venipuncture from the umbilical cord and, following umbilical arterial catheter placement into ascending aorta, perinatal asphyxia was induced prior to delivery through 15 or 18 min cord clamping. The population concerning this article comprised 20 primates undergoing umbilical cord clamping and four controls. After delivery, all the subjects in need were stabilized applying standardized resuscitation principles. Apgar scores, vital signs, and serial laboratory parameters were collected, and blood was drawn a second time from the umbilical catheter at 5 min of age. Samples were analyzed by two-dimensional gas chromatography coupled to time-of-flight mass spectrometry (GC × GC–TOFMS), and processed through PCA and Fisher ratio analysis, leading to the identification of 14 metabolites responsible for the profile differences between pre- and post-asphyxia. Comparing these groups, an elevation was observed in the concentration of all the 15 metabolites, *i.e*., succinic acid, lactate, glutamate, 9*H*-purine, malate, glycerol, glucose-1-phosphate, arachidonic acid, leucine, creatinine, fructose, myo-inositol, butanoic acid, and pantothenic acid. Following the comparison between the pre- and post-asphyxia metabolic variation of pathological and control samples, 10 of the 14 metabolites were found to be significative. Therefore, although lactate and creatinine were confirmed as biomarkers for asphyxia, the most sensitive metabolite was succinic acid. Moreover, also malate and arachidonic acid levels increased. Such finding was explained for malate by fast oxidative stress effects on the Krebs cycle, while for arachidonic acid by either the disruption of the cell membrane or by the downstream interruption of its metabolic pathways. Nevertheless, differences between animal subjected to 15 rather than 18 min asphyxia were not observable.

In 2012, Beckstrom *et al*. [23] focused on the metabolic analysis of blood samples during the transition between foetus and neonate, being the first work covering such changes. During the experimental procedures, samples were collected from six primates delivered at a gestation comparable to a 36th week of human gestation, at nine time points within the first 3 days after birth (0, 0.012, 1, 3, 6, 12, 24, 48, and 72 h). Due to Dunnett’s test, 23 significant metabolites, belonging to five different classes, were identified as the most susceptible to changes during this period of time (Table 1). In particular, while most of the metabolites increased over time, compounds such as lactate, proline, hippuric acid, and benzoic acid decreased. Furthermore, α-ketoglutaric acid, fumaric acid, malate, and succinyl-CoA, belonging to Krebs cycle, showed significant variations within this window of time. This was explained by the rapid change in oxygen exposure that characterizes the body transition from a more anaerobic metabolism to a preponderant aerobic metabolism through the activation of Krebs cycle. Interesting, oppositely to Krebs intermediates, lactate levels raised in the first 5 min after birth to successively decrease 1 h of age.

Therefore, these works support the involvement of redox mechanisms into pathological and development dynamics, confirming previous studies on molecular and genomic levels. However, also in this case, a larger number of subjects is required to validate the employment of such metabolites as health status or maturity biomarkers.

### 3.3. Piglet Model

To our knowledge, piglet has been the only species studied both through NMR and MS platforms, analysing both urine and plasma. As discussed by Saugstad *et al*. [26] the use of oxygen in the resuscitation therapies for newborns [4] should be tested and revaluated. Animal studies suggested that room air reduced the formation of free radicals produced by higher oxygen concentrations [24], and hyperoxia approach may induce oxidative stress and multiorgan damage [27]. In order to assess how different oxygen concentration may affect newborn subjects, two groups designed their works to study this topic. In 2010, Solberg and co-workers [24] tested the effects of hypoxaemia and different resuscitation approaches on a total of 27 new-born piglets. Indeed, femoral artery blood samples were collected before and after the induction of hypoxaemia, and after resuscitation through 15 min of 21% or 100% oxygen followed by 45 min of 21% oxygen or 60 min of 100% oxygen flow. In order to detect a larger range of metabolites, both Flow Injection Analysis (FIA)-MS/MS and LC-MS/MS techniques were applied. Chemometric approaches highlighted 45 compounds and 11 metabolites ratios as discriminating in case of hypoxaemia. In the majority of the subjects, lactate, α-ketoglutaric acid, succinic acid, and fumarate levels increased as a consequence of hypoxaemia, and lowered after resuscitation. However, while lactate decreased regardless the oxygen concentration applied, the other metabolites levels lowered at different rates, showing a faster reaction with 21% oxygen flow. Moreover, as consequence of 100% oxygen approach, lanosterol and oxysterols (24*S*-hydroxycholesterol and 25-hydroxycholesterol) levels were found significantly increased, suggesting inefficient cholesterol synthesis and neuronal damage, respectively. Accordingly to previous reports, this study found decrements in free carnitine and decadienyl-L-carnitine, and increments in long chain acylcarnitines when severe, prolonged hypoxaemia occurred. These data suggested an incomplete fatty acid oxidation, which leads to the affection of normal mitochondrial activity, neurological injury, and enhanced oxidative stress. Furthermore, multivariate analysis identified 57 compounds and 16 ratios as significant for the response to resuscitation treatments. In particular, due to the reduction of metabolic noise, the correlation between Ala/Branched Chain Amino Acids (BCAA) or Gly/BCAA ratios and duration of hypoxaemia was positively predicting, especially in combination with other metabolites such as succinic acid and propionyl-L-carnitine. These latter parameters may constitute an improvement compared to the current lactate and pH-based ones, which are affected by other metabolic compensatory mechanisms, because ratios were reliable beyond the first 15–30 min of hypoxaemia. More recently, the experiment performed by Fanos *et al.* [25] in 2014 was reported. In particular, their work tested the use of four different oxygen concentrations (18%, 21%, 40%, and 100%) analyzing urine samples from 38 piglets through a NMR platform. Such samples were collected at two different time points; before hypoxia induction, and after reoxygenation and 30 min stabilization of the animals. The study showed that 21% oxygen flow was the most efficient resuscitation strategy in terms of survival, rapidity of resuscitation and restore of the metabolic profile. Bioinformatics analysis highlighted the effects on different pathways for each different oxygen concentration applied giving a deeper insight on the response dynamic. The employment of 18% oxygen emphasized carbohydrate metabolism, 21% cellular function maintenance, whereas 40% and 100% were characterized by free radical processes (supporting the Saugstad thesis). In particular, using 21% oxygen concentration, the discriminating metabolites were glucose, lactate, alanine, glycerate, pyruvate, malonate, glycine, succinic acid, 3-methyladenine, acetylglycine, glutaconic acid, 4-hydroxyphenylpyruvate, and 3-hydroxymethyl glutarate, while higher oxygen approaches showed notable presence of creatinine, urea, citric acid, tartaric acid, ethanol, glucose, and indoxyl sulphate. Therefore, the combination of these two works gave a broad view on the metabolic changes in piglets in both blood and urine medium.

Dealing with the diagnostic capacity of metabolomics, an article was reported in 2013 by Skappak and co-workers [9] on the analysis of 1–3 days old male Yorkshire-Landrace piglets’ urine samples. The 32 subjects were anesthetized and divided into two groups, one under normal ventilation condition (*n* = 15), and one undergoing 2 h hypoxia (*n* = 17) followed by reoxygenation procedure. Urine samples were collected at the end of the experiment using bladder transcutaneous angiocatheter and analysed through NMR spectroscopy. Interestingly, while standard physiological parameters such as arterial oxygen saturation, pH, mean arterial pressure (MAP), and CO returned to normal non-hypoxic values after 4 h recovery, metabolomics approach was able to distinguish hypoxic *vs.* non-hypoxic subjects, hence proving to be a more accurate strategy. Indeed, 1-methylnicotinamide, 2-oxoglutarate, alanine, asparagine, betaine, citric acid, creatine, fumarate, hippurate, lactate, *N*-acetylglycine, *N*-carbamoyl-β-alanine, and valine levels were found to be predictive of the insult in 84% of the cases. Notably, no single, independent metabolite was suitable for such diagnosis, confirming the strength of the holistic approach.

Despite the considerable amount of data and information regarding the effects and mechanisms of hypoxia insult, which constitute a solid starting point, these findings need to be confirmed with metabolomics analysis on human subjects in order to accurately describe and manage such occurrence.

### 3.4. Human Studies

Metabolomics studies on infant and adult subjects, performed within the 2006–2014 window of time, are summarised in Table 2. The first metabolomic study on neonatal hypoxia was reported by Chu *et al*., in 2006 [28], who analyzed urine samples by GC-MS. The examined population was formed by 24 infants admitted to Neonatal Intensive Care Unit (NICU) and 13 controls. The 24 neonates were further classified as having a good perinatal outcome unless they died (PND = PeriNatal Death) or developed seizures within 7 days from delivery. Thirteen subjects were classified as “good outcome” (no HIE or PND) and eleven as “poor outcome” (eight HIE and three PND). Sample treatment included a solvent extraction (ethyl acetate) after an oximation step to analyse only the carboxylic acid fraction. After derivatization with BSTFA, the GC-MS analysis identified 130 metabolites, and hierarchical clustering analysis allowed for the classification into three different profiles: controls, NICU-good-outcome, and NICU-Poor-outcome. Among the entire set of carboxylic acids, eight molecules resulted statistically significant. Indeed, ethyl malonate, 3-hydroxy-3-methylglutarate, 2-hydroxy-glutarate, and 2-oxoglutarate were more abundant in urines of infants with “good outcome” compared to those with “poor outcome”, while glutarate, methylmalonate, 3-hydroxybutyrate, and orotate were more abundant in infants with “poor outcome”.

Two other papers were published by the same group, which set similar experimental design in both cases. In particular, they analyzed the umbilical cord blood by means of LC-MS/MS [29], and ^1^H-NMR spectroscopy [30]. Walsh *et al*. [29] examined 71 infants divided into those with HIE (31), and those with biochemical or clinical risk of asphyxia without encephalopathy (40). All cases were compared to a control cohort of infants (71) matching both infant and maternal demographic parameters, including gestational age, gender, birth weight and centile, method of delivery, maternal ethnicity, maternal age, and maternal body mass index (BMI). Given the sporadic nature and lack of antenatal warnings for perinatal asphyxia, it may be difficult to recruit and collect suitable samples in infants. This has led to multiple studies of neonatal asphyxia including a heterogeneous population of mixed gestational age, and broad inclusion parameters.

**Table 2:** Metabolomic analysis on human models.

Method	Biofluid	Aim	Population	Discriminant metabolites	Reference
GC-MS Organic acids	Urine	Discrimination of different outcomes	13 good-outcome *vs.* matched controls 11 poor-outcome *vs.* matched controls	**Increase(good > poor)**: ethylmalonate, 3-hydroxy-3-methylglutarate, 2-hydroxyglutarate, 2-oxoglutarate **Decrease** **(poor > good):** glutarate, methylmalonate, 3-hydroxybutyrate, orotate	Chu *et al*., 2006 [28]
LC-MS/MS	Umbilical cord blood	Discrimination between asphyxia and HIE occurrence	40 Asphyxia *vs.* matched controls	lysoPC a C16:0, PC aa C34:1, PC aa C36:4, PC aa C38:4, PC aa C38:5, taurine ^a^	Walsh *et al*., 2012 [29]
			31 HIE *vs.* matched controls	alanine, asparagine, isoleucine, methionine, phenylalanine , proline, tyrosine, valine, PC ae C38:4	
^1^H-NMR	Umbilical cord blood	Discrimination between asphyxia and HIE occurrence	34 Asphyxia *vs.* matched controls	3-hydroxybutyrate, acetone, alanine, betaine, choline, creatine, creatinine, glucose, glycerol, isoleucine, lactate, leucine, myo-inositol, *O*-phosphocholine, phenylalanine, pyruvate, succinate, valine	Reinke *et al*., 2013 [30]
			25 HIE *vs.* matched controls	alanine, choline, creatine, glycerol, isoleucine, lactate, leucine, methionine, myo-inositol, phenylalanine, pyruvate, succinate, valine	
LC-TOF/MS	Urine	Differences between pre- and post-hypoxia	6 adults	**Increase**: 1-methyladenosine, uric acid, xanthine, carnitine, propanoyl carnitine **Decrease**: 3-indoleacetic acid, glutamine, glutamic acid, succinic acid	Bih-Show 2014 [ 31]

^a^ PC aa C34:1 denotes a phosphatidylcholine with 34 carbons in the two fatty acid (aa) bonded to the glycerol moiety and a single double bond in one of them.

Recognising the complex physiology and multiple potential aetiologies of this disease, this group attempted to remove as many confusing variables as possible, limiting their analyses to term infants with the injury carefully defined using both modified Sarnat score and electroencephalogram (EEG). The modified Sarnat score was the primary means used to categorise grade of injury and the EEG was used to validate these findings. The cord blood was analyzed with an assay kit (Biocrates AbsolutIDQTM p180 kit), allowing for both sample derivatization and MS data analysis, and an Applied Biosystems/MDS Sciex QTrap LC-MS/MS system. This platform enabled the indentification and quantification of 148 metabolites belonging to five different classes; acylcarnitines, glycerophospholipids, sphyngolipids, amino acids and biogenic amines.

The PLS-DA analysis of mass data allowed for the identification of three class of metabolites as significant in the discrimination between the examined populations: acylcarnitines, glycerophospholipids and amino acids. 14 metabolites (13 acylcarnitines + amino acid leucine) differ significantly in both comparisons asphyxia/control and HIE/control; nine metabolites (eight amino acids + one glycerophospholipid) were significant only for HIE/control; and six metabolites (five glycerophospholipids + the biogenic amine taurine) were significant for asphyxia/control. In this study, acylcarnitines were raised in infants with both asphyxia and HIE relative to controls, while amino acids were predominantly raised in those with HIE. Walsh’s work demonstrates the potential for the metabolic signature present in the umbilical cord blood to distinguish injury severity, and therefore the possibility of directing the need for treatment. The multivariate PLS-DA models showed a clear metabolites associations with both asphyxia and HIE. The PLS-DA model discriminating HIE from all other outcomes indicated that the biomarker signature for HIE has the potential to predict severity of insult rather than a yes/no binary outcome. In particular, the more severe the modified Sarnat score the more accurate the classification. Using robust data mining and modelling techniques, they have shown that the metabolite profile in the cord blood at birth, representing the latent systems-wide interaction in the metabolome, is sufficient to produce a robust predictive model for presence of encephalopathy at 24 h of age with an area under ROC curve (AUC) of 0.93.

Reinke *et al*. [30] conducted a study on an infant population analogous to the one described above. In fact, 59 cases were divided in 25 infants with HIE, including 13 mild, six moderate, and six severe cases, and 34 infants who were asphyxiated but did not present clinical neurological signs. Since in the previous work central energy metabolites such as organic acids and carbohydrates were not identified, an NMR platform was used. One-dimensional ^1^H-NMR spectra of umbilical cord blood samples were acquired using a 600 MHz Varian Inova spectrometer. Blood samples were deproteinized using perchloric acid, pH was adjusted (it is not reported to which value) and volume was diluted to 190 μL with water, using 10 μL of a 5 mM solution of 2,2-dimethyl-2-sila-3,3,4,4,5,5-hexadeutero-pentanesulfonic acid (DSS) as internal standard. Metabolite identification and quantification in the acquired NMR spectra were accomplished using the 600 MHz database provided in Chenomx NMR Suite Professional software v5.1 (Chenomx Inc., Edmonton, AB, Canada). In this way, 37 metabolites belonging to the classes of organic acids and alcohols, amino acids, ketones, biogenic amines, together with glucose and urea, were identified and quantified. Through multivariate analysis, 18 metabolites were found to be significantly different between asphyxia *vs.* matched controls and 13 significantly different between HIE *vs.* matched controls; while 12 of these metabolites were common to both groups.

A recent work by Bih-Show *et al*. [31] studied the effect of hypoxia on six adults. They were subjected, at different time, to both severe (12% O_2_ concentration) and moderate hypoxia (15%) for 2 h. Urine samples were collected before and after hypoxia induction and were analyzed by LC-MS. PLS-DA on mass data allowed for the identification of 26 metabolites belonging to four different classes (nucleic acid, amino acid, carnitines, and miscellaneous organic compounds) as significant discriminants for pre- and post-hypoxia events. Further analysis highlighted 1-methyladenosine, uric acid, xanthine, carnitine, propanoylcarnitine, indoleacetic acid, glutamine, glutamate, and succinic acid as dose-depending metabolites. Therefore, it showed the involvement of lipids and energy metabolisms. Interestingly, subjects’ metabolic profiles related to mild hypoxia were not similar; however, such difference was less pronounced in severe hypoxia cases.

## 4. Conclusions

At present, the literature does not count a large number of articles on metabolomic analysis of perinatal asphyxia. The objectives of such works cover a broad range of interest as well as a diverse type of species under study. Indeed, the majority of the research has been performed through animal models, while only three articles investigated infants within the period of time from 2006 to 2013. Rodent model employed brain extracts, from slices or whole organs, to validate the application of hypothermic therapy. Although the experimental design introduced several variables to the studies, metabolomics was able to discriminate among subjects that received different treatments (hypothermia, delayed hypothermia, normothermia), supporting hypothermia beneficial effects, and proposing the application of new biomarkers such as glutamine together with other important metabolites. Monkey models, through blood and umbilical cord blood analysis, highlighted the differences between pre- and post-asphyxia condition (induced by umbilical cord clamping), and in the metabolic transition from foetus to newborn. These experiments identified discriminating metabolites and suggested the involvement of redox processes. Regarding piglet model studies, the main issues discussed were the response to different oxygen concentration for resuscitation therapies, and the recognition of hypoxic cases from controls. In the former case, not only was possible to describe the metabolites trends before and after hypoxemia occurrence, but also to highlight the metabolic pathways affected by each concentration, supporting the use of 21% oxygen concentration as a more appropriate resuscitation treatment. The smaller number of human model studies may be explained both by the reduced availability of subjects, and by the impossibility to design and test risky treatment on infants. Nevertheless, Chu’s analysis was able to assess the severity of the newborns, while Walsh’s and Reinke’s method could successfully separate pathological (asphyxia or HIE) from healthy subjects. Lou’s study on healthy adults, simulating hypoxic conditions, found discriminant metabolites for the recognition of mild from severe hypoxia. Unfortunately, only a few articles were able to underline a metabolic pathway affected by hypoxia condition. From all the works described above, the involvement of the Krebs cycle seems to be a common finding, although other molecules were significant for discrimination. Supporting this observation, the increase of branched chain amino acids may be caused by the necessity of an alternative energy source for muscles and brain. Another interesting feature, common to Solberg’s piglet and Walsh’s human studies who both employed LC-MS/MS, is the key role played by long chain acylcarnitines. In fact, the reduced rate for β-oxidation of long chain acyl-CoA due to hypoxia occurrence, seems to induce the accumulation of their precursors (acylcarnitines). Notably, among the metabolites proposed as biomarkers, no one could be singly employed for the characterisation of the clinical status, demonstrating the necessity of an holistic approach. In addition, a recent review by Denihan *et al.* [32] reported cases of perinatal asphyxia studies, focusing on pathological aspects, result validation and patient recruitment. Given the diversity of the objectives discussed in these articles it is possible to understand how versatile this discipline is. In fact, although metabolomics is still a growing discipline and more studies are necessary for the validation for these results, it proved to possess the potentiality to become a useful tool for the assessment and the monitoring in perinatal clinical practice.

## List of Abbreviations

ACSF: Artificial CerebroSpinal Fluid
NMR: Nuclear Magnetic Resonance
PCA: Principal Component Analysis
PLS-DA: Partial Least Square Discriminant Analysis
PCr: PhosphoCreatine
ELISA: Enzyme-Linked ImmunoSorbent Assay
PCho: PhosphoCholine
OGD: Oxygen–Glucose Deprivation
GC×GC–TOFMS: Gas Chromatography coupled to Time-Of-Flight Mass Spectrometry
HIE: Hypoxic Ischemic Encephalopathy
OXPHOS: OXidative PHOSphorylation
ROS: Reactive Oxygen Species
FIA: Flow Injection Analysis
LC: Liquid Chromatography
MAP: Mean Arterial Pressure
BCAA: Branched Chain Amino Acids
Ala: Alanine
GC: Gas Chromatography
NICU: Neonatal Intensive Care Unit
PND: PeriNatal Death
BMI: Body Mass Index
BSTFA: Bis(trimethylsilyl)trifluoroacetamid
EEG: ElectroEncephaloGram
CoA: Coenzyme A
AUC: Area Under ROC Curve
DSS: 2,2-Dimethyl-2-Sila-3,3,4,4,5,5-hexadeuteropentane sulfonic acid
